# Physiological Properties of Cholinergic and Non-Cholinergic Magnocellular Neurons in Acute Slices from Adult Mouse Nucleus Basalis

**DOI:** 10.1371/journal.pone.0011046

**Published:** 2010-06-10

**Authors:** Tristan Hedrick, Jack Waters

**Affiliations:** Department of Physiology, Feinberg School of Medicine, Northwestern University, Chicago, Illinois, United States of America; The Research Center of Neurobiology-Neurophysiology of Marseille, France

## Abstract

**Background:**

The basal forebrain is a series of nuclei that provides cholinergic input to much of the forebrain. The most posterior of these nuclei, nucleus basalis, provides cholinergic drive to neocortex and is involved in arousal and attention. The physiological properties of neurons in anterior basal forebrain nuclei, including medial septum, the diagonal band of Broca and substantia innominata, have been described previously. In contrast the physiological properties of neurons in nucleus basalis, the most posterior nucleus of the basal forebrain, are unknown.

**Methodology/Principal Findings:**

Here we investigate the physiological properties of neurons in adult mouse nucleus basalis. We obtained cell-attached and whole-cell recordings from magnocellular neurons in slices from P42-54 mice and compared cholinergic and non-cholinergic neurons, distinguished retrospectively by anti-choline acetyltransferase immunocytochemistry. The majority (70–80%) of cholinergic and non-cholinergic neurons were silent at rest. Spontaneously active cholinergic and non-cholinergic neurons exhibited irregular spiking at 3 Hz and at 0.3 to 13.4 Hz, respectively. Cholinergic neurons had smaller, broader action potentials than non-cholinergic neurons (amplitudes 64±3.4 and 75±2 mV; half widths 0.52±0.04 and 0.33±0.02 ms). Cholinergic neurons displayed a more pronounced slow after-hyperpolarization than non-cholinergic neurons (13.3±2.2 and 3.6±0.5 mV) and were unable to spike at high frequencies during tonic current injection (maximum frequencies of ∼20 Hz and >120 Hz).

**Conclusions/Significance:**

Our results indicate that neurons in nucleus basalis share similar physiological properties with neurons in anterior regions of the basal forebrain. Furthermore, cholinergic and non-cholinergic neurons in nucleus basalis can be distinguished by their responses to injected current. To our knowledge, this is the first description of the physiological properties of cholinergic and non-cholinergic neurons in the posterior aspects of the basal forebrain complex and the first study of basal forebrain neurons from the mouse.

## Introduction

Cholinergic drive to the forebrain plays a role in arousal and attention and is essential for many learning and memory tasks [1], [2]. Dysfunction of this input has been tied to pathophysiological conditions such as depression and Alzheimer's disease [3]–[5]. Forebrain cholinergic drive is provided by the basal forebrain, a series of nuclei distributed through the ventral aspect of the forebrain that includes medial septum, the diagonal band of Broca, substantia innominata and nucleus basalis magnocellularis [6], [7]. Each of these nuclei contains a heterogeneous collection of neurons, including both cholinergic and non-cholinergic projecting magnocellular neurons. Neurons in nucleus basalis project diffusely to cortex, sending cholinergic, GABAergic (γ-aminobutyric acid), and possibly glutamatergic axons to the entire cortical mantle [8], [9].

The electrophysiological properties of cholinergic and non-cholinergic neurons in medial septum, the diagonal band of Broca and substantia innominata have been studied previously in dissociated culture and in brain slices [10]–[27]. Comparable studies of neurons in more posterior aspects of the basal forebrain have not been performed. Furthermore, previous studies have mostly used early postnatal tissue from rat and guinea pig. The physiological properties of basal forebrain neurons have not been studied in the mouse.

Here we characterize the membrane and firing properties of cholinergic and non-cholinergic neurons in nucleus basalis of the adult mouse, using cell-attached and whole-cell recordings to characterize neurons in acute slices and retrospective anti-choline acetyltransferase (ChAT) immunocytochemistry to identify cholinergic neurons. We show that cholinergic and non-cholinergic neurons have different action potential waveforms, after-spike potentials and spiking rates during constant current injection.

## Methods

### Ethics statement

All experiments and procedures were approved by the Northwestern University Institutional Animal Care and Use Committee (IACUC).

### Slice preparation

P42-P54 C57BL-6 mice were anaesthetized using an interperitoneal injection of 120 mg/kg ketamine and 50 mg/kg xylazine in phosphate-buffered saline (PBS): 75 mM Na_2_HPO_4_, 25 mM NaH_2_PO_4_, pH 7.4, and transcardially perfused with ice cold sucrose-artificial cerebrospinal fluid (ACSF): 85mM NaCl, 2.5mM KCl, 1.25mM NaH_2_PO_4_, 20mM NaHCO_3_, 10mM HEPES, 25mM glucose, 75mM sucrose, 0.5mM CaCl_2_, 4mM MgCl_2_, pH 7.3, gassed with 95% O_2_/5% CO_2_. The brain was quickly removed and 300 µm coronal slices were cut in sucrose-ACSF using a vibrating microslicer (Vibratome, St. Louis MO). Slices were held in sucrose-ACSF at 37°C for 5–15 minutes and thereafter at room temperature in ACSF: 125mM NaCl, 2.5 KCl, 1.25mM NaH_2_PO_4_, 20mM NaHCO_3_, 5mM HEPES, 25mM glucose, 1mM CaCl_2_, 2mM MgCl_2_, pH 7.3, gassed with 95% O_2_/5% CO_2_.

### Electrophysiology

Slices were transferred to the stage of an upright microscope (Olympus BX51) and continuously perfused with ACSF gassed with 95% O_2_/5% CO_2_ and warmed to 37°C. Nucleus basalis was identified by comparison to an atlas of the mouse brain [28] and published anti-ChAT immunocytochemistry [6]. Recordings were obtained under infra-red difference interference contrast optics from magnocellular neurons ∼50–75 µm below the surface of the slice. Whole-cell recording pipettes were 4–8 MΩ when filled with intracellular solution: 135mM K gluconate, 4mM KCl, 10mM HEPES, 10mM Na_2_-phosphocreatine, 4mM Mg-ATP, 0.3mM Na_2_-GTP, 0.2% (w/v) biocytin, 10 µM alexa 488, pH 7.3. Signals were recorded with an Axoclamp-2A amplifier (Molecular Devices, Sunnyvale CA), National Instruments A-to-D boards and Labview software written by JW (National Instruments, Austin TX).

After recording, images of the alexa-filled neuron were acquired using 2-photon microscopy for morphological analysis. The slice was then fixed overnight in 4% (w/v) paraformaldehyde in PBS and stored for up to 1 month at −20°C in 30% (v/v) ethylene glycol and 30% (v/v) glycerol in phosphate buffer for later immunocytochemical analysis.

For cell-attached recordings, pipettes were filled with ACSF supplemented with 100 µM alexa 488 dextran (10,000 MW). After obtaining a loose seal (20–200 MΩ), we recorded spontaneous activity for several minutes. We then imaged the region near the tip of the pipette to confirm that alexa had not entered the neuron, indicating that the cell membrane had not been ruptured during recording. In neurons which were not spontaneously active, we evoked action potentials by current injection through the recording pipette (0.2–100 nA; 300 ms pulses). Where action potentials did not occur spontaneously and could not be evoked, we were unable to confirm that we were recording from a neuron and we therefore discarded the recording.

### Immunocytochemistry

Slices were resectioned into 75 µm-thick sections using a Vibrating microslicer, blocked for one hour at room temperature in 1% bovine serum albumin and 1% triton in PBS and incubated overnight at room temperature in an anti-ChAT polyclonal primary antibody (1∶200 dilution of AB144P; Millipore, Billerica MA). Sections were then incubated for two hours at room temperature with a Cy3-conjugated secondary antibody (Jackson Immunoresearch, West Grove PA) and Cy2-conjugated streptavidin (Jackson Immunoresearch, West Grove PA).

**Figure 1:**
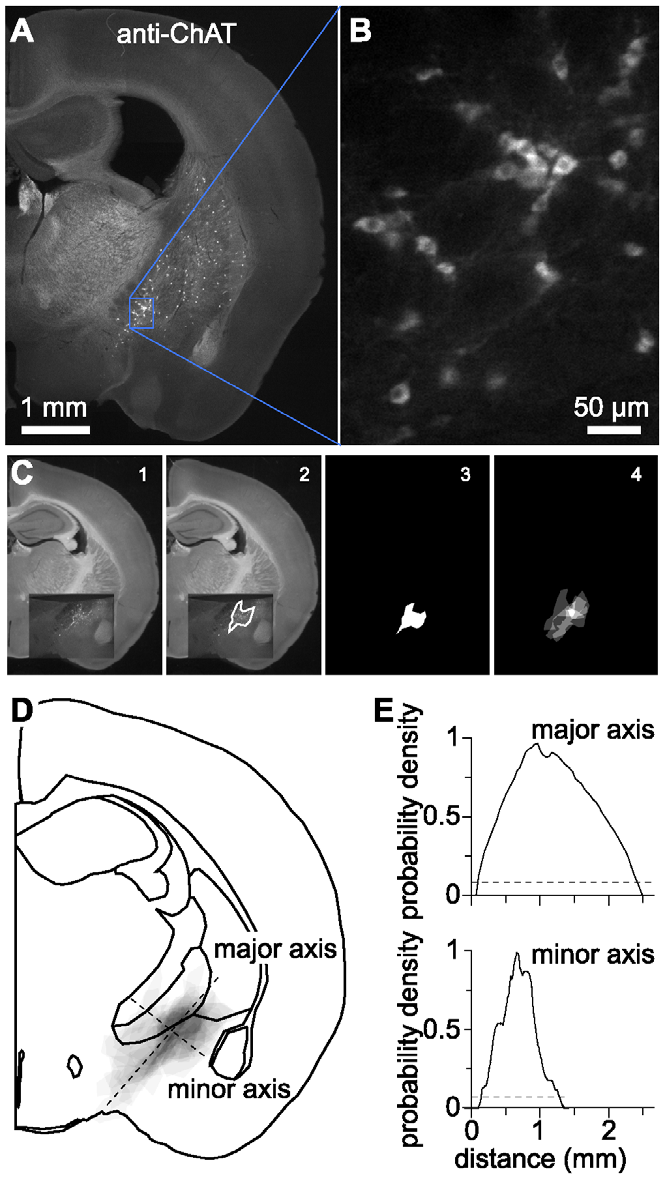
Location and dimensions of nucleus basalis. (**A**) Widefield image of anti-ChAT immunofluorescence of a coronal section, approximately 1mm posterior to bregma, with prominent anti-ChAT staining in nucleus basalis and caudate putamen. (**B**) A sub-region of the section in A, showing ChAT-immunoreactive somata in nucleus basalis. (**C**) Illustration of the method used to describe the location of nucleus basalis. To pool the locations of nucleus basalis from multiple animals, for each section we first overlaid the anti-ChAT image onto a brightfield image of a representative coronal section (panel 1). This aligned all anti-ChAT images to the common brightfield image. For each section we manually drew an outline around all ChAT^+^ somata in nucleus basalis (panel 2) and converted this outline to a binary mask (panel 3). These masks were averaged to give a probability density distribution for nucleus basalis (panel 4). (**D**) The probability density distribution was then aligned with a schematic of the coronal slice for display purposes. To calculate the dimensions of nucleus basalis we measured the probability density along major and minor axes of the approximately elliptical probability density distribution (dashed lines). (**E**) A threshold of 10% of the peak intensity (dashed line) in each axis gave dimensions for nucleus basalis of 2.52 mm in the ventromedial-dorsolateral (major) axis and 1.12 mm in the dorsomedial-ventrolateral (minor) axis.

Nucleus basalis was identified in each section as a dense collection of ChAT^+^ somata in the ventral portion of the slice, approximately 0–500 µm posterior to bregma [6], [28]. To compare recording locations and the position of nucleus basalis in different slices we calculated the mean location of nucleus basalis in our slices (figure 1C–E). For each slice we created a binary mask corresponding to nucleus basalis by drawing an outline around nucleus basalis in the anti-ChAT fluorescence image. Masks from all slices were aligned to a brightfield image of a coronal section. After alignment, masks were collated into a single stack and averaged by calculating a mean intensity projection.

### Analysis

Analysis was performed using routines written by JW in IgorPro v6.0 (Wavemetrics, Lake Oswego OR). Physiological properties were measured in bridge mode, using 1 ms or 300 ms square current injections. V-I relationships were derived from the steady-state voltage during 300 ms current steps and were fit with the following relationship [29]:
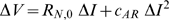
where R_N_ is the resting input resistance (slope at I = 0)

C_AR_ is a quadratic coefficient which describes the curvature of the V-I relationship

Voltage sag was estimated as the ratio of peak and steady-state voltages during the 300 ms current step. The membrane time constant was determined by fitting an exponential to the rising phase of the voltage response during the current step.

Action potential threshold was defined as the point at which the first temporal derivative of the voltage (dV/dt) first exceeded 55 mV/ms. Action potential amplitude was measured from threshold. Action potential half width was measured at half the amplitude. Action potential rise and decay times were measured as the time intervals between 10 and 90% of the voltage difference between threshold and peak. Fast after-hyperpolarization (fAHP) amplitude was measured after the first spike during 300 ms current injection. fAHP amplitude was defined as the voltage difference between threshold and the minimum voltage within 10 ms after the peak of the action potential. To measure the after-depolarization (ADP) and slow after-hyperpolarization (sAHP), single action potentials were evoked with 1ms current injections. ADP amplitude was defined as the voltage difference between the peak of the fAHP and the maximum voltage between fAHP and sAHP. sAHP amplitude was defined as the voltage difference between the resting membrane potential and the most negative voltage between the ADP and the point of return to the resting membrane potential. sAHP latency was calculated as the time difference between the peak of the action potential and the peak of the sAHP. The 90% decay time of the sAHP was the time difference between the peak of the sAHP and the point at which the sAHP had decayed to 10% of its peak amplitude.

## Results

### Location and identification of nucleus basalis

To characterize the physiological properties of neurons in nucleus basalis we obtained recordings from nucleus basalis in acute coronal slices from adult mice. After recording, we fixed slices overnight and labeled cholinergic neurons by anti-ChAT immunocytochemistry (figure 1A,B). As in previous studies, nucleus basalis appeared as an approximately elliptical region of dense somatic anti-ChAT immunoreactivity, extending a mean distance of 2.52 mm in the ventromedial-dorsolateral (major) axis and 1.12 mm in the dorsomedial-ventrolateral (minor) axis (figure 1D–E).

‘Nucleus basalis’ refers to a collection of brain regions, but the term has not been used consistently by different authors. Some authors have included substantia innominata and the magnocellular preoptic nucleus within nucleus basalis, in which case these regions form the anterior limit of nucleus basalis [6], [7], [10], [30]. Here we studied more posterior aspects of nucleus basalis, approximately corresponding to the anterior part of the Ch4p region as defined by Mesulam [30], and use the term ‘nucleus basalis’ to refer to only these nuclei, which are posterior to the anterior commissure and in approximately the same coronal plane as the anterior extent of the optic tract.

### Few magnocellular neurons in nucleus basalis are spontaneously active

**Figure 2:**
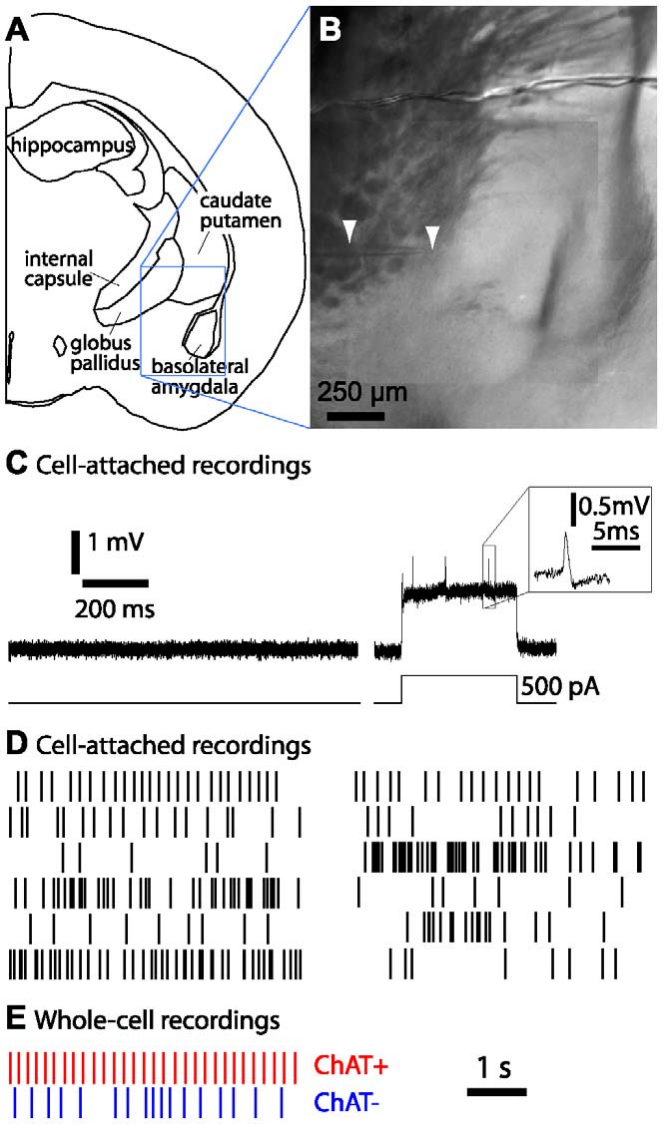
Cell-attached recordings and spontaneous firing activity. (**A**) Schematic illustration of a coronal slice approximately 1mm posterior to bregma, based on the atlas of Franklin and Paxinos (2008) [25]. (**B**) Brightfield image of a slice showing the region around nucleus basalis during recording from a neuron in nucleus basalis. Arrowheads mark the recording pipette. (**C**) Example of a cell-attached recording from a neuron which did not exhibit spontaneous activity. Current injection (500 pA, 300 ms) evoked action potentials. Inset: action potential waveform. (**D**) Raster plots showing 10 seconds of irregular firing in 12 spontaneously firing neurons recorded in the cell-attached configuration. Each vertical line denotes the timing of an action potential and neurons are arranged in two columns, each containing the plots for 6 neurons. (**E**) Raster plots from two spontaneously firing neurons in the whole-cell configuration. The neurons were identified as ChAT+ (red) and ChAT− (blue) neurons by retrospective immunocytochemistry.

We obtained cell-attached recordings from magnocellular neurons in nucleus basalis (figure 2A,B). Only 12 of 40 neurons (30%) fired spontaneously. When a neuron was silent at rest, we injected current through the recording pipette to evoke spikes (figure 2C). No neurons fired bursts of action potentials, either spontaneously or during current injection, as reported in some recordings from more anterior regions of basal forebrain [14]–[21], [26]. In our recordings, spontaneously active neurons fired irregularly at a mean frequency of 2.7±1.1 Hz (range: 0.3 to 13.4 Hz; figure 2D).

Spontaneous activity was similar in whole-cell recordings (figure 2E), in which we retrospectively identified cholinergic and non-cholinergic neurons (see below). 2 of 9 ChAT^+^ and 6 of 30 ChAT^−^ neurons (approximately 20% of each group) fired spontaneously at rest in irregular patterns at frequencies of 3 Hz and 2–11.5 Hz, respectively. In these recordings spontaneous firing was eliminated with ≤20 pA holding current.

### Identification of cholinergic and non-cholinergic neurons

**Figure 3:**
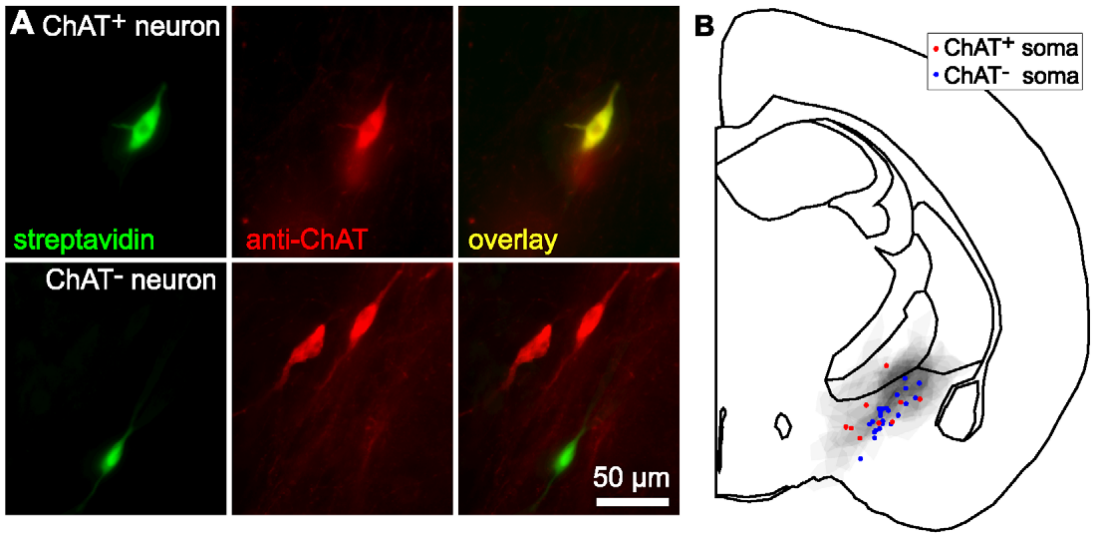
Identification of cholinergic and non-cholinergic neurons in nucleus basalis. (**A**) Examples of retrospective streptavidin (green) and anti-ChAT (red) fluorescence for two recorded neurons. In one recording the streptavidin-positive soma co-labels with anti-ChAT. Anti-ChAT fails to label the other neuron, although nearby cells are ChAT^+^, indicating that the anti-ChAT staining was successful in this slice. (**B**) Summary schematic showing the locations of nucleus basalis and of all recordings. Red and blue markers indicate the somatic locations of recordings from ChAT^+^ and ChAT^−^ neurons, respectively. Grey shading indicates the probability distribution for nucleus basalis (see Methods section and figure 1).

During whole-cell recording we filled neurons with biocytin through the recording pipette, fixed and resectioned slices after recording and retrospectively identified cholinergic neurons by anti-ChAT immunocytochemistry, using fluorescently-tagged streptavidin to label the neuron from which we had recorded (figure 3A). All recordings were from within nucleus basalis (figure 2B).

Neurons from which we had recorded were considered cholinergic if the intensity of anti-ChAT fluorescence at the soma was at least 75% of the intensity of other ChAT-immunoreactive (ChAT^+^) neurons in the same slice. Neurons were considered non-cholinergic if the intensity was less than 25% of that of ChAT^+^ neurons in the slice. Neurons with somatic intensities between 25 and 75% of ChAT^+^ neurons were not classified and were excluded from further analysis. Of 50 recordings (from 29 mice) we identified 9 ChAT^+^ neurons and 30 ChAT^−^ neurons, leaving 11 neurons unclassified.

### Subthreshold characteristics of ChAT^+^ and ChAT^−^ neurons are similar

**Figure 4:**
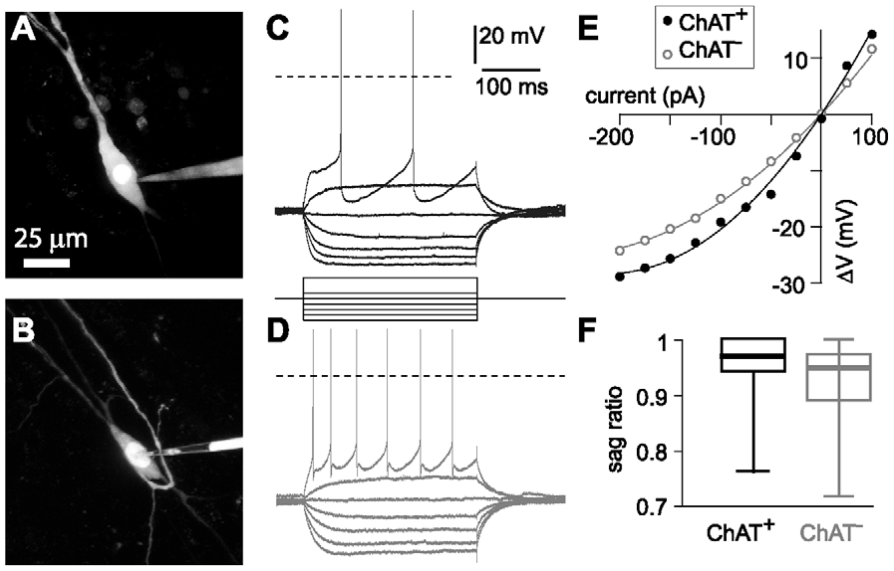
Examples of whole-cell recordings and subthreshold characteristics. (**A and B**) Maximum intensity projections of 2-photon image stacks acquired during recording from two neurons, one ChAT^+^ (A) and one ChAT^−^ (B). In each, the recording pipette is visible to the right of the soma. (**C and D**) Voltage responses to −200, −150, −100, −50, 0, 50 and +200 pA current steps for 300ms. Dashed lines indicate 0 mV. C is from the ChAT^+^ neuron in A; D from the ChAT^−^ neuron in B. (**E**) Voltage-current curves for the ChAT^+^ (black, filled symbols) and ChAT^−^ (open, grey symbols) neurons in A and B, both showing anomalous rectification at potentials hyperpolarized to rest. (**F**) Sag ratio (steady state / peak voltage) during 300 ms, −200 pA current steps in ChAT^+^ and ChAT^−^ neurons.

All of our recordings were from magnocellular neurons. We measured somatic size from fluorescence images acquired during recording, filling neurons with Alexa 488 via the recording pipette (figure 4A,B). Somatic size was similar for ChAT^+^ and ChAT^−^ neurons (ChAT^+^ neurons were 35.3×15.8 µm; ChAT^−^ neurons were 29.7×13.5 µm; table 1).

**Table 1:** Electrophysiology of ChAT^+^ and ChAT^−^ magnocellular neurons in adult mouse nucleus basalis.

	ChAT+			ChAT−			Mann-Whitney	t-Test
	mean	sem	n	mean	sem	n	P value	P value
soma diameter, major axis (µm)	35.3	3.5	8	29.7	1.6	28	0.1068	0.1053
soma diameter, minor axis (µm)	15.8	1.7	8	13.5	0.7	28	0.1564	0.1230
resting membrane potential (mV)	−55.4	4.8	7	−56.8	1.5	24	0.8717	0.7024
resting input resistance (MΩ)	251.4	17.7	9	281.4	21.6	30	0.6054	0.4594
C_AR_ (MΩ/nA)	357.0	100.7	9	519.5	69.0	30	0.2642	0.2353
membrane time constant (ms)	27.7	6.8	6	38.1	5.9	28	0.6436	0.4278
sag ratio	0.98	0.02	9	0.93	0.01	30	0.0169	0.0559
rheobase (pA)	25.6	8.8	9	48.1	5.5	30	0.9817	0.8181
AP threshold (mV)	−31.5	2.8	9	−37.6	1.6	30	0.0272	0.0114
AP amplitude (mV)	64.0	3.4	9	74.9	2.0	30	0.0080	0.0096
AP half width (ms)	0.52	0.04	9	0.33	0.02	30	<0.0001	<0.0001
AP 10–90% rise time (ms)	0.24	0.02	9	0.16	0.01	30	<0.0001	<0.0001
AP 10–90% decay time (ms)	0.49	0.03	9	0.29	0.02	30	0.0001	0.0002
AP amplitude accommodation 3/1	0.85	0.03	8	0.92	0.02	29	0.0094	0.0469
AP amplitude accommodation 10/1	N/A	N/A	N/A	0.75	0.03	29		
fAHP amplitude (mV)	16.6	3.5	9	19.8	1.3	30	0.5823	0.2661
ADP amplitude (mV)	0.1	0.1	9	2.9	0.4	30	0.0003	0.0004
sAHP amplitude (mV)	13.3	2.2	9	3.6	0.5	30	<0.0001	<0.0001
sAHP latency (ms)	34.9	3.0	9	30.5	3.1	24	0.4546	0.4164
sAHP 90% decay time (ms)	0.24	0.03	9	0.18	0.03	24	0.0224	0.3062

Previous authors have suggested that in anterior regions of the basal forebrain, such as medial septum and diagonal band, cholinergic and non-cholinergic neurons have different subthreshold membrane properties and spiking patterns. To compare the physiological properties of neurons in nucleus basalis we injected hyperpolarizing and depolarizing current steps (figure 4C,D). ChAT^+^ and ChAT^−^ neurons had similar resting membrane potentials (−55 mV and −57mV) and input resistances (250 and 280 MΩ, respectively; table 1). At subthreshold potentials, both ChAT^+^ and ChAT^−^ neurons displayed anomalous rectification, with no significant difference in the curvature of the V-I relationship (figure 4E and table 1). Membrane sag, which often indicates the presence of h-type non-specific cation conductance [31], [32], was negligible in ChAT^+^ neurons and significantly larger, but still small in ChAT^−^ neurons (figure 4C,D,F and table 1).

### ChAT^+^ neurons fire at lower frequencies than ChAT^−^ neurons during tonic depolarization

**Figure 5:**
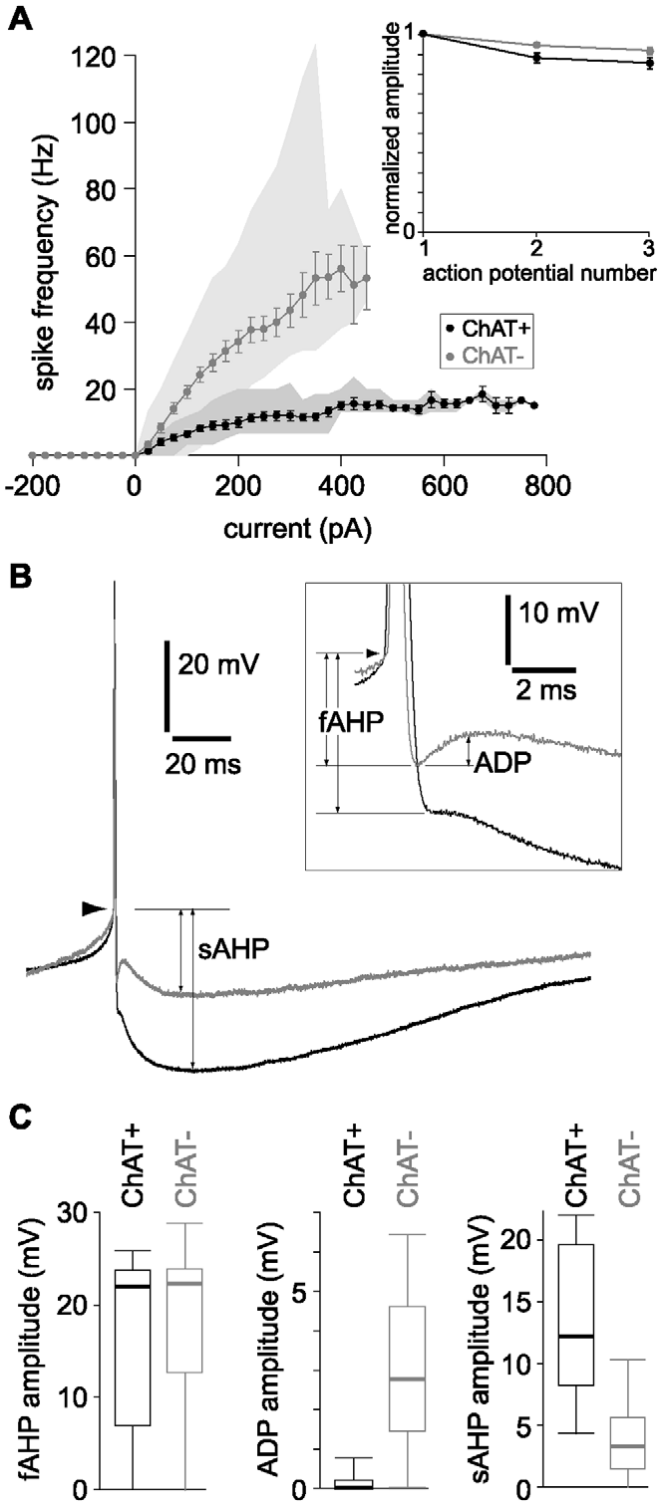
Comparison of firing rates and after-spike potentials. (**A**) Spike frequency as a function of injected current for ChAT^+^ (black symbols) and ChAT^−^ neurons (grey symbols). The shaded areas denote the full range of each population (n = 9 ChAT^+^ and 28 ChAT^−^ neurons). Inset: Spike amplitudes during trials with only three action potentials. ChAT^+^ (black symbols) and ChAT^−^ neurons (grey symbols) displayed weak spike amplitude accommodation. (**B**) Example action potentials from a ChAT^+^ (black) and a ChAT^−^ (grey) neuron, illustrating the differences in after-spike potentials. Traces were aligned to the threshold (arrowhead). Inset: Same action potentials on an expanded time scale, clearly showing the fAHP and, in the ChAT^−^ neuron, the ADP. (**C**) Plots showing the amplitudes of fAHP, ADP and sAHP for ChAT^+^ (black) and ChAT^−^ (grey) neurons. n = 9 ChAT^+^ and 30 ChAT^−^ neurons.

A prominent difference between ChAT^+^ and ChAT^−^ neurons was the lower spiking frequency of ChAT^+^ neurons during depolarizing current injections (figure 4C,D). With increasing current injections the spiking rates of ChAT^+^ neurons increased to a plateau frequency of ∼20 Hz, with no rise in spike rate with current injections greater than approximately 400pA (figure 5A). Similar current injections evoked spiking at up to ∼120 Hz in ChAT^−^ neurons and spike rate failed to reach a plateau frequency with increasing current injection. The difference in spike rate was sufficiently pronounced that the neurons could readily be identified as ChAT^+^ or ChAT^−^ from their firing rates alone.

Both ChAT^+^ and ChAT^−^ neurons displayed weak amplitude accommodation during sustained spiking (figure 5A, inset). During action potential trains at 10 Hz, the normalized action potential amplitude (normalized to the first action potential) was 0.85±0.03 for ChAT^+^ neurons and 0.92±0.02 for ChAT^−^ neurons (table 1; P = 0.0094, Mann-Whitney test).

The contrasting spike rates of ChAT^+^ and ChAT^−^ neurons likely result principally from differences in their after-spike potentials. After repolarization of the spike, ChAT^+^ neurons display a fast after-spike hyperpolarization (fAHP; 16.5±3.5 mV), negligible spike after-depolarization (ADP; 0.13±0.1 mV) and a pronounced slow AHP (sAHP; 13.3±2.2 mV). ChAT^−^ neurons display a fAHP of similar amplitude (19.8±1.3 mV), followed by a larger, but brief ADP (2.9±0.4 mV) and a much smaller sAHP (3.6±0.5 mV) than ChAT^+^ neurons (figure 5B,C and table 1).

Of the 11 cells that we were unable to classify as ChAT^+^ or ChAT^−^, 3 displayed the firing frequency and after-spike potentials of a cholinergic neuron, while the firing frequencies and after-spike potentials of the other 8 indicate that they were non-cholinergic neurons.

### ChAT^+^ neurons have broader action potentials than ChAT^−^ neurons

**Figure 6:**
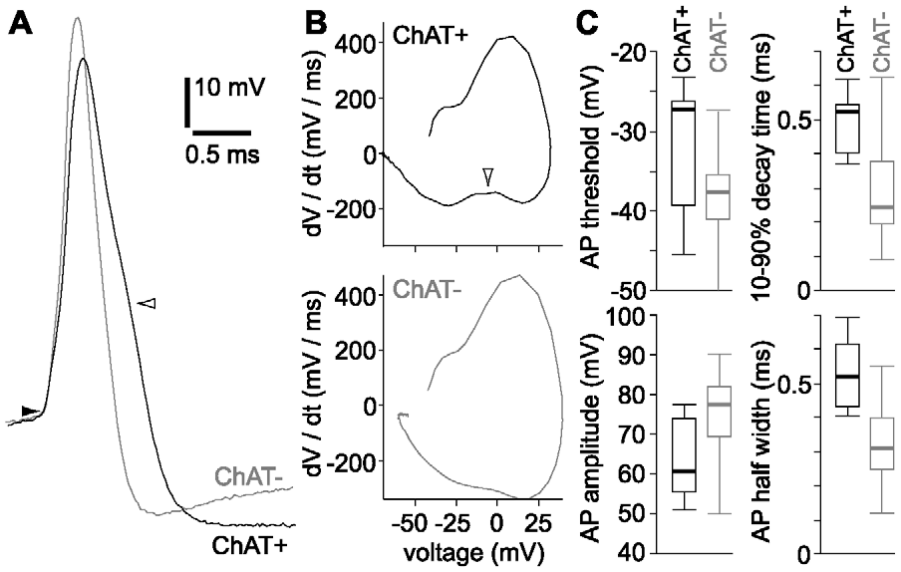
Comparison of action potential waveforms. (**A**) Example action potentials from a ChAT^+^ (black) and a ChAT^−^ (grey) neuron, illustrating the difference in spike waveform. Spikes are aligned to threshold (filled arrowhead). The ChAT^+^ neuron has a broader action potential with a shoulder on the decaying phase (open arrowhead). (**B**) The shoulder (open arrowhead) is clearly visible in the phase plot for ChAT^+^ action potential: the shoulder reduces the maximum rate of decay of the action potential. (**C**) Threshold, amplitude, half width and decay time for action potentials in ChAT^+^ (black) and ChAT^−^ (grey) neurons. n = 9 ChAT^+^ and 30 ChAT^−^ neurons.

ChAT^+^ and ChAT^−^ neurons also have different action potential waveforms. Action potentials in ChAT^+^ neurons were of smaller amplitude and were broader than action potentials in ChAT^−^ neurons (figure 6). The broad action potential in ChAT^+^ neurons resulted, in part, from a shoulder on the decaying phase of the action potential (figure 6A). This shoulder was clearly visible in phase plots of action potentials from ChAT^+^, but not ChAT^−^ neurons (figure 6B). Action potential threshold, amplitude, half width, rise time and decay time all differed significantly between ChAT^+^ and ChAT^−^ neurons (table 1), but action potential waveform alone was insufficient to identify a neuron as cholinergic or non-cholinergic, there being considerable overlap in these parameters between the two populations (figure 6C).

## Discussion

We have demonstrated that magnocellular cholinergic neurons in nucleus basalis in acute slices from adult mice differ from non-cholinergic magnocellular neurons in several respects, including spiking rates, spike waveforms and after-spike potentials. Of these properties, the spiking rate was the most distinctive feature: firing rates were sufficiently different that a cholinergic neuron could be identified from its slow firing rate during somatic current injection.

### Comparison with neurons in other basal forebrain nuclei

Most previous studies of the physiology of basal forebrain neurons have concentrated on the medial septum and diagonal band of Broca from young guinea pigs or rats. Cholinergic neurons in medial septum and diagonal band show anomalous rectification [16], [17], [24]–[26], [33]; a lack of membrane sag [16], [24], [26]; a broader, slower-decaying action potential than non-cholinergic neurons [16]; a more prominent sAHP than non-cholinergic neurons [13], [16]–[18], [24]; and relatively low spike rates [13], [16], [18], [24], [26].

The cholinergic neurons of more posterior basal forebrain nuclei have received less attention. Neurons in substantia innominata and magnocellular preoptic nucleus may be similar in many respects to the neurons in medial septum and diagonal band [26] although it has also been suggested that these neurons have remarkably different properties, firing action potentials in regularly-occurring bursts resulting from tetrodotoxin-insensitive, broad depolarizations in both young guinea-pig slices [10], [20] and in the adult rat *in vivo* [34], [35].

Here we present the first study of the cellular properties of neurons in more posterior aspects of nucleus basalis. We found that both cholinergic and non-cholinergic neurons fired irregularly-spaced action potentials during constant suprathreshold depolarizing current injections: we observed no examples of burst firing. Our results indicate that the sub- and suprathreshold electrophysiological properties of cholinergic neurons in nucleus basalis are similar to those of cholinergic neurons in medial septum and diagonal band. Indeed, it seems likely that cholinergic neurons throughout much of the basal forebrain share similar electrophysiological properties, with the possible exception of neurons in substantia innominata and the magnocellular preoptic nucleus [10], [20], [34], [35].

### Prevalence of cholinergic neurons in nucleus basalis

Many of the projecting neurons in nucleus basalis are cholinergic, but there are disparate estimates of the proportions of neurons that are cholinergic and non-cholinergic. Retrograde tracing techniques in combination with acetylcholinesterase histochemistry and anti-ChAT immunocytochemistry indicate that between 40 and 90% of projecting neurons are cholinergic in rhesus monkey and rat, respectively [30], [36].

In our recordings from adult mice, only ∼23% of magnocellular neurons (9 of 39) were ChAT^+^. Some *in vivo* studies have also reported that ∼25% of magnocellular neurons were cholinergic in substantia innominata and magnocellular preoptic nucleus [34], [35]. In contrast, 40–50% of magnocellular neurons were found to be cholinergic in previous studies of anterior basal forebrain nuclei from young rodents [16], [24], [26]. The lower incidence of cholinergic neurons in our slices than in previous studies and than expected from anatomical studies of nucleus basalis could reflect species differences, a difference between anterior and posterior basal forebrain nuclei or could be a selection bias resulting from the slicing process. Cholinergic neurons are particularly prone to excitotoxicity [37], [38] and there might therefore be a preferential loss of cholinergic neurons during slicing. We have found that cutting slices in a high-sucrose ACSF with a reduced calcium/magnesium ratio enhanced the probability of recording cholinergic neurons in our slice preparation (data not shown). It seems unlikely that the slicing procedure biases recordings to a sub-population of cholinergic neurons, but *in vivo* recordings from nucleus basalis will be required to unequivocally answer this question.

### Physiological significance of the low spikes rates and pronounced sAHP in cholinergic neurons

Our results might suggest that the pronounced sAHP of cholinergic basal forebrain neurons causes them to spike at lower frequencies *in vivo* than non-cholinergic basal forebrain neurons, limiting release of ACh in the cortex. However, our recordings report the properties of neurons *in vitro*, where there is little ongoing synaptic activity or neuromodulatory drive. The spiking rates of cholinergic neurons in substantia innominata and magnocellular preoptic nucleus are affected by neuromodulators, including noradrenaline [39], histamine [40], serotonin [41] and neurotensin [42]. These neuromodulators act via a variety of mechanisms, including inhibition of the sAHP [43]. The pronounced sAHP may therefore provide a mechanism by which the spiking rates of cholinergic neurons in nucleus basalis may be readily switched by neuromodulators between fast- and slow-spiking modes. Changes in neuromodulatory drive to nucleus basalis with behavioral state may thus provide course control of cholinergic drive to the neocortex with changing behavior. There is evidence for modulation with behavioral state *in vivo*: the activity patterns of neurons in substantia innominata and magnocellular preoptic nucleus change during the sleep-wake cycle in the rat [44]–[46] and neurotensin can induce similar changes by facilitating bursting of cholinergic neurons [47]. It is likely that other neuromodulators, perhaps acting via inhibition of the sAHP can play similar roles during behavior.

How the properties of cholinergic neurons in nucleus basalis change *in vivo*, with behavior and altered neuromodulatory drive, remains to be determined. Our results describe the baseline state of neurons in nucleus basalis, to which properties *in vivo* can be compared as they become available.
